# Loneliness and self-harm in adolescents during the first national COVID-19 lockdown: results from a survey of 10,000 secondary school pupils in England

**DOI:** 10.1007/s12144-022-03651-5

**Published:** 2022-09-15

**Authors:** Galit Geulayov, Karen Mansfield, Christoph Jindra, Keith Hawton, Mina Fazel

**Affiliations:** 1 Centre for Suicide Research, Department of Psychiatry, University of Oxford, Warneford Hospital, Oxford, UK; 2 Section of Child and Adolescent Psychiatry, Department of Psychiatry, University of Oxford, Warneford Hospital, Oxford, UK; 3 Institute for Educational Quality Improvement, Humboldt University, Berlin, Germany; 4 Oxford Health NHS Foundation Trust, Oxford, UK; 5 Oxford University Hospitals NHS Foundation Trust, Oxford, UK

**Keywords:** Loneliness, Self-harm, Adolescents, COVID-19, Pandemic

## Abstract

Adolescents’ loneliness and self-harm have received considerable attention during the COVID-19 pandemic with concerns that the socioecological changes taking place would contribute to an escalation of both loneliness and self-harm. However, empirical evidence is scant. We estimated the prevalence of loneliness and self-harm in adolescent school pupils and investigated the association of loneliness and change in loneliness during the UK’s first lockdown with self-harm during lockdown in a cross-sectional school survey (OxWell) involving 10,460 12–18-year-olds from south England. Loneliness was measured with four items. Self-harm was ascertained through a detailed questionnaire. The prevalence of loneliness and self-harm were estimated applying post-stratification weights to account for differences between the study sample and the target population. The associations between indicators of loneliness and self-harm were examined using mixed effect models. 1,896 of 10,460 adolescents (18.1%) reported feeling lonely ‘often’ (weighted proportion 16.8%). 3,802/10,460 (36.4%; weighted proportion 35%) felt more lonely since lockdown. Self-harm during lockdown was reported by 787/10,460 adolescents (7.5%; weighted proportion 6.7%). Controlling for confounders, adolescents who reported feeling lonely ‘often’ [adjusted odds ratio (aOR) 2.8, 95% CI 2.1–3.9, *p* < 0.0001] or ‘sometimes’ (aOR 2.2, 95% CI 1.5–3.2, *p* < 0.0001) were more likely to self-harm during lockdown relative to adolescents who reported ‘never’ or ‘hardly ever’ feeling lonely. Exacerbation in loneliness during lockdown was associated with an increase in the odds of self-harm during lockdown. Loneliness, heightened loneliness and self-harm were common during lockdown and closely linked. It is important to support schools in address loneliness and self-harm as part of efforts to improve well-being as the long tail of the pandemic continues to impact on child and adolescent mental health. Understanding how loneliness and self-harm may co-vary could be important for future self-harm reduction strategies in young persons.

**Supplementary Information:**

The online version contains supplementary material available at 10.1007/s12144-022-03651-5.

## Introduction

From the earliest stages of the COVID-19 pandemic there have been concerns that the profound changes to adolescents’ social and educational milieu would negatively impact their health and well-being. It has been suggested that loneliness would intensify and that self-harm could increase (Carr et al., 2021; Jollant et al., 2021; Loades et al., 2020; O'Connor et al., 2020). Loneliness has been defined as “a subjective, unwelcome feeling of lack or loss of companionship”. It happens when there is “a mismatch between the quantity and quality of social relationships that we have, and those that we want.” (Perlman and Peplau, 1981). There have been growing concerns about the prevalence of loneliness among adolescents and its impact on their health and well-being, even before the global coronavirus pandemic began. In one pre-pandemic survey, 10% of 16–24 year-olds reported that they felt lonely ‘often’ or ‘always’, which was more than in adults (Department for Digital Culture Media & Sport, 2020). Findings from another survey showed that in 2018 11% of 10 to 15 year-olds reported ‘often’ feeling lonely (Office for National Statistics, 2018a). Since the start of the COVID-19 pandemic, adolescents have had to deal with a significant disruption to their physical, academic, and social routine, with the majority experiencing diminished in-person social contacts (Loades et al., 2020). It has been suggested that loneliness would be exacerbated. However, evidence is scant. In one retrospective survey involving 12–17 year-olds from Scotland, the prevalence of loneliness was reported to have increased from 9% before to 28% following lockdown (Generation Scotland, 2020). In the 2020 YouGov survey, 41% of the children and young persons surveyed reported that they were lonelier than they had been before lockdown (Barnardo’s, 2020). There is some evidence from other countries on the negative impact of the pandemic disease containment measures on adolescents’ loneliness (Ellis et al., 2020) although findings from a prospective study involving Australian youth (Houghton et al., 2022) and findings from a Norwegian study using repeated cross-sectional surveys (Myhr et al., 2021) have shown no increase in loneliness in relation to school closure and disease containment measures earlier in the pandemic relative to pre-pandemic measures.

Self-harm (intentional self-poisoning or injury) is relatively common in adolescents in both clinical settings and in the community (Geulayov et al., 2018; Hawton et al., 2002; Madge et al., 2008; McMahon et al., 2014; Moran et al., 2012). Self-harm in adolescence is linked to myriad adverse outcomes, including premature mortality (Geulayov et al., 2019; Hawton et al., 2012, 2020a, 2020b; Morgan et al., 2017; Olfson et al., 2018) and poorer psychosocial outcomes such as depression and substance misuse (Borschmann et al., 2017; Mars et al., 2014). It is also often repeated, which further increases suicide risk (Geulayov et al., 2019). Moreover, the negative impact of adolescent self-harm can extend to their family members (Ferrey et al., 2016) and may also influence the risk of self-harm by their peers (Hawton et al., 2020a, 2020b). Some pre-pandemic reports from high-income nations have suggested an increased rate of self-harm in adolescents in recent years. In the UK, for example, incidence rates of presentations to clinical services following self-harm in 13–16-year-old females increased by 68% between 2011 and 2014 (Morgan et al., 2017). Less is known about changes in self-harm in the community, primarily because many adolescents do not present to clinical services (Geulayov et al., 2018). A study reporting on two different community-based cohorts suggested an increase in self-harm in 14 year-olds from 12% in 2005 to 14% in 2015 (Patalay & Gage, 2019). Furthermore, a recent systematic review involving 12–18-year-olds (Gillies et al., 2018) reported a lifetime prevalence of self-harm of 17%, although prevalence varied considerably (range 4% to 39%). Past year self-harm was recorded at 13%. The three UK-based studies in this review suggested that approximately 14% of adolescents had self-harmed over their lifetime (during 2001–2012).

Loneliness and thwarted social ties have been linked to a person’s risk of self-harm and suicide. Concepts such as connectedness, belonging and social integration are central features in theories of suicidal behaviour (Joiner, 2005; Klonsky & May, 2015; Van Orden et al., 2010). Previous research has shown that individuals reporting more intense feelings of loneliness are more likely than their peers to report suicidal thoughts and behaviours (Calati et al., 2019; McClelland et al., 2020) although evidence from studies involving children and adolescents is mixed (McClelland et al., 2020). However, relationship problems (with peers and family members) have been reported in many studies as major risk factors for self-harm in children and adolescents (Evans et al., 2004). Published data about the relationship between loneliness and self-harm pre-date the pandemic. Furthermore, little is known about how changes in loneliness might be related to self-harm. The aim of this study was to estimate the prevalence of loneliness and self-harm in a large and diverse community-based sample of 10,460 12–18-year-old adolescents after the first few months of the COVID-19 pandemic and to investigate the associations of loneliness and changes in loneliness during lockdown with self-harm. Loneliness and self-harm were both self-reported.

## Method

### Participants and procedure

The OxWell School Survey is a repeated cross-sectional, self-report survey on children and adolescents’ mental health and well-being, commencing in 2019. We used data on adolescents attending school years 8 to 13 (aged 12–18 years) from secondary educational institutions in England, recruited through local authorities. All state-maintained schools, academies and independent schools as well as further education colleges (FECs) were eligible to take part. Pupils were invited to take part through their school using a parental opt-out process (Mansfield et al., 2021).

We report on data collected in 2020. 14,352 pupils from 91 schools across 11 local authorities (Figure S1) in the south of England enrolled to take part. The 2020 survey was completed during June-July either on school premises or from home due to partial school closures during the first COVID-19 UK national lockdown which commenced on 23^rd^ March 2020.

### *Measures*

Loneliness was measured with four items. Two items from the UCLA-3 Loneliness Scale (Russell, 1996; Russell et al., 1980): 1. “How often do you feel that you have no one to talk to?” 2.”How often do you feel left out?” And a direct question about loneliness, 3. “How often do you feel lonely?” All three items had three-response categories: *Never/hardly ever, sometimes,* and *often*. These were scored 1, 2, and 3, respectively. We used the versions of questions adapted and validated for use with children and adolescents (Office for National Statistics, 2018b). The direct question was treated as a stand-alone item, while the two items from the UCLA-3 Loneliness Scale were used to derive a summary score- only calculated for respondents who answered both these items. The score ranged between 2 and 6, with a higher score indicating greater loneliness. To assess change in perceived loneliness in relation to national lockdown, we used an item which enquired about the extent to which loneliness had changed over lockdown. It was measured on a scale from 0 to 100, presented to participants with five-point anchor points: *Much less, Slightly less, Same, Slightly more, Much more*. A higher score indicated a greater sense of loneliness during lockdown.

Self-harm refers to any act of non-fatal intentional self-poisoning or self-injury, irrespective of the nature or the degree of suicidal intent (Hawton et al., 2003). Self-poisoning includes the intentional ingestion of any drug where the amount exceeds that prescribed or the ingestion of non-ingestible substances, overdoses of ‘recreational drugs’, and severe alcohol intoxication where the individual intended to harm themselves. Self-injury is defined as any injury that has been intentionally self-inflicted. Information on self-harm was collected through a detailed questionnaire, which was based on the method used in the Child and Adolescent Self-harm in Europe (CASE) study (Madge et al., 2008). *Lifetime self-harm*: intentional self-poisoning or self-injury which had occurred at any point prior to the survey. We used two questions to ascertain lifetime self-harm (Table S1). For respondents who endorsed item *1* (‘*Ever self-harmed’*) their free-text item (item 8) describing their act of self-harm was reviewed by two researchers (GG and ES) independently. They were classified with ‘lifetime self-harm’ if their described act (item 8) met the study criteria (Hawton et al., 2002). Otherwise they were not considered to have self-harmed. *Past year self-harm*: intentional self-poisoning or self-injury which occurred in the 12 months before the survey. Respondents who endorsed item *1* (‘*Ever self-harmed’)*, had self-harmed within the past year (items *4*, ‘*Last self-injury*’ and/or *7, ‘Last self-poisoned’*), and who described a valid method of self-harm (item *8*) were considered to have self-harmed in the past year. *Self-harm during lockdown*: In the UK the first lockdown (‘stay-at-home’ order) commenced on 23^rd^ March 2020. Self-harm during lockdown was recorded as intentional self-poisoning or self-injury which occurred between 23^rd^ March and the date of survey (June-July 2020). Respondents were not presented with specific dates but the UK national lockdown coincided with the closure of schools to all but certain groups of pupils. Respondents who endorsed item *1* ‘*Ever self-harmed* ‘ and item *2* ‘*Ever self-injured*’ (from ‘*Once or twice’* to ‘*Daily’*) and item *3 ‘Self-injured during lockdown’* (from ‘*Once or twice*’ to ‘*Most days’*) OR if they endorsed items *1* and *5 ‘Ever self-poisoned’* (‘*Yes’*) and *6 ‘Self-poisoned during lockdown’* (‘*Yes’*) were classified as having self-harmed during lockdown (provided their described act of self-harm met the study criteria). All others were treated as not having self-harmed during lockdown (Table S1).

For information about symptoms of depression and anxiety we used the Revised Children’s Anxiety and Depression Scales (RCADS-25) (Chorpita et al., 2000; Ebesutani et al., 2012). We included participants who provided a response to at least 80% of the RCADS items. We derived validated t-scores for depression and anxiety (Child Outcomes Research Consortium (CORC), 2020). We also created two binary groups with RCADS t-scores ≥ 70 indicating ‘probable depression/anxiety’, while a score < 70 was categorised as ‘no depression/anxiety’. We further used the item: “Have you ever received any mental health support? Yes/No” to identify pupils with a history of mental health difficulties. Gender, year group (a proxy for age), respondent birth place (“Were you born in the UK?” *Yes/No/Rather not say*), socioeconomic deprivation (eligibility for free school meals and household food insecurity) were self-reported. Free school meals (FSM) is an official indicator of socioeconomic disadvantage in children and adolescents used in the UK.

#### Ethical approval:

The study received ethical approval (Ref R62366/RE0010) from the University of Oxford Medical Sciences Division Research Ethics Committee (MSDREC).

#### Consent to participate

Pupils under 16 years gave active assent to participate; those 16 years and over consented to the study.

### Statistical analysis

Respondents’ characteristics and prevalence of loneliness and self-harm are presented as unweighted and weighted proportions with corresponding 95% confidence interval.

We used mixed-effects logistic models to examine the associations between indicators of loneliness and self-harm. Self-harm during the first UK lockdown was treated as the dependent variable and random intercepts were used at the local authority, school, and year group levels, to account for clustering. We ran the following models: 1) comparing the odds of self-harm during lockdown in relation to the level of loneliness and; 2) in relation to the UCLA two-item score; 3) the odds of self-harm during lockdown in relation to change in level of loneliness during lockdown. Gender, age (year group), respondent birth place, socioeconomic deprivation (eligibility for free school meals and household food insecurity), and mental health problems (symptoms of depression and anxiety, ever receiving mental health support) were added as covariates in the final models. These covariates were selected a priori as they have been previously shown to be associated with the risk of self-harm and with indicators of loneliness (Loades et al., 2020).

#### Weights

Due to possible differences between the sample surveyed in the OxWell 2020 survey and the target population (all those attending the identified schools), we applied post-stratification weights. Non-response may have arisen from multiple sources (i.e. differences in propensity to be involved by local authorities, schools and/or pupils). We calculated post-stratification weights to reduce possible non-response bias using raking and auxiliary information for a subset of demographics that could be matched with the UK Office for National Statistics (ONS) Census data for the participating counties. Weights were derived using regional census data including information on Type of school (Independent versus other i.e. state primary/secondary); Gender (Male/Female); English as first language (we used a proxy of child and both parents born in UK); Age; Index of Multiple Deprivation.

Analyses were carried out using Stata 14.2.

## Results

The sample selection process is displayed in Figure S1. Table 1 shows the characteristics of the analytic sample by gender. Of the 10,460 respondents, 6,604 (63.1%) were female (weighed proportion: 53.8%). Over 50% were pupils attending years 8–9 (12–14-year-olds).

**Table 1:** Characteristics of the analytic sample, unweighted and weighted proportion with 95% confidence interval, by gender

	Total			Males			Females		
	N	Unweighted % (95% CI)	Weighted % [95% CI]^a^	N	Unweighted % (95% CI)	Weighted % [95% CI]^a^	N	Unweighted % (95% CI)	Weighted % [95% CI]^a^
*N* = 10,460				3,856	36.9 (36.0–37.9)	46.2 [45.2–47.2]	6,604	63.1 (62.2–64.1)	53.8 [52.8–54.9]
Sociodemographic characteristics									
School year									
Year 8–9 (age 12–14 years)	5,367	51.3 (50.0–52.3)	53.3 [52.2–54.2]	2,044	53.0 (51.4–54.6)	54.6 [53.0–56.2]	3,323	50.3 (51.4–54.6)	52.0 [50.8–53.3]
Year 10–11 (age 14–16 years)	3,266	31.2 (30.1–32.1)	29.6 [28.7–30.5]	1,098	28.5 (27.0–30.0)	27.3 [25.9–28.8]	2,168	32.8 (31.7–34.0)	31.6 [30.4–32.7]
Year 12–13 (age 16–18 years)	1,827	17.5 (16.8–18.2)	17.2 [16.4–17.9]	714	18.5 (17.3–19.8)	18.1 [16.9–19.3]	1,113	16.9 (16.0–17.8)	16.4 [15.5–17.3]
Pupil birth place									
Non-UK	1,295	12.4 (11.8–13.0)	17.5 [16.6–18.4]	503	13.1 (12.0–14.1)	18.3 [17.0–19.8]	792	12.0 (11.2–12.8)	16.7 [15.7–17.8]
UK born	9,082	86.8 (86.2–87.5)	81.7 [80.8–82.6]	3,320	86.1 (85.0–87.2)	80.8 [79.3–82.2]	5,762	87.3 (86.4–88.0)	82.5 [81.4–83.5]
Unknown	83	0.8 (0.6–1.0)	0.8 [0.6–1.0]	33	0.9 (0.6–1.2)	8.4 [0.6–1.2]	50	0.8 (0.6–1.0)	0.8 [0.6–1.0]
Parents birth place									
Non-UK	3,857	36.9 (36.0–37.8)	40.7 [39.7–41.7]	1,470	38.1 (36.6–39.7)	41.9 [40.3–43.6]	2,387	36.2 (35.0–37.3)	39.6 [38.4–40.1]
UK born	6,414	61.3 (60.4–62.3)	57.6 [56.6–58.6]	2,318	60.1 (58.6–61.7)	56.4 [54.8–58.1]	4,096	62.0 (61.8–63.2)	58.6 [57.4–59.8]
Unknown	189	1.8 (1.6–2.1)	1.7 [1.5–2.0]	68	1.8 (1.4–2.2)	1.7 [1.3–2.1]	121	1.8 (1.5–2.2)	1.8 [1.5–2.2]
Free school meals									
No	7,876	75.3 (74.5–76.1)	74.6 [73.8–75.5]	2,794	72.5 (71.0–73.8)	72.2 [70.7–73.7]	5,082	77.0 (75.9–78.0)	76.7 [75.7–77.8]
Yes	793	7.6 (7.1–8.1)	7.7 [7.1–8.2]	308	8.0 (7.2–8.9)	7.9 [7.1–8.9]	485	7.3 (6.7–8.0)	7.4 [6.8–8.1]
Not known	1,791	17.1 (16.4–17.9)	17.7 [16.9–18.5]	754	19.6 (18.3–20.8)	19.8 [18.6–21.2]	1,037	15.7 (14.8–16.6)	15.8 [14.9–16.8]
Ever experienced food poverty									
No	9,136	87.3 (86.7–88.0)	87.4 [86.8–88.1]	3,362	87.2 (86.1–88.2)	87.3 [86.1–88.3]	5,774	87.4 (86.6–88.2)	87.6 [86.7–88.4]
Yes^b^	929	8.9 (8.4–9.4)	8.8 [8.2–9.4]	334	8.7 (7.8–9.6)	8.6 [7.8–9.6]	595	9.0 (8.3–9.7)	8.9 [8.2–9.6]
Not known	395	3.8 (3.4–4.2)	3.8 [3.4–4.2]	160	4.1 (3.6–4.8)	4.1 [3.5–4.8]	235	3.6 (3.1–4.0)	3.5 [3.1–4.0]
Mental health									
Symptoms of depression (RCAD_D), mean (95% CI)^**c**^	10,383	50.6 (50.3–50.9)	49.8 [49.5–50.0]	3,818	45.9 (45.5–46.3)	45.8 [45.4–46.2]	6,565	53.3 (53.0–53.7)	53.2 [52.8–53.5]
Symptoms of anxiety (RCAD_A), mean (95% CI)^**c**^	10,383	49.8 (49.6–50.1)	49.1 [48.9–49.4]	3,818	46.0 (45.6–46.4)	46.0 [45.6–46.3]	6,565	52.0 (51.7–52.3)	51.8 [51.5–52.2]
Ever received mental health support									
No	7,822	74.8 (73.9–76.6)	76.3 [75.5–77.2]	3,153	81.8 (80.5–83.0)	82.0 [80.7–83.2]	4,669	70.7 (69.6–71.8)	71.5 [70.4–72.6]
Yes	2,569	24.6 (23.7–24.4)	23.0 [22.2–23.9]	680	17.6 (16.5–18.9)	17.4 [16.2–18.7]	1,889	28.6 (27.5–29.7)	27.8 [26.8–28.9]
Not known	69	0.7 (0.5–0.8)	0.6 [0.5–0.8]	23	0.6 (0.4–0.9)	0.6 [0.4–0.9]	46	0.7 (0.5–0.9)	0.7 [0.5–0.9]
School characteristics									
Rural/urban									
Rural	1,698	16.2 (15.5–17.0)	15.4 [14.7–16.2]	537	13.9 (12.9–15.1)	13.6 [12.5–14.7]	1,161	17.6 (16.7–18.5)	17.0 [16.1–18.0]
Urban	8,762	83.8 (83.0–84.5)	84.6 [83.8–85.3]	3,319	86.1 (84.9–87.1)	86.4 [85.3–87.5]	5,443	82.4 (84.9–87.1)	83.0 [82.0–83.9]
Funding source									
State funded	9,158	87.6 (86.9–88.2)	87.2 [86.6–87.9]	3,241	84.1 (82.9–85.2)	84.3 [83.1–85.4]	5,917	89.6 (88.8–90.3)	89.8 [89.0–90.5]
Independent	967	9.2 (8.7–9.8)	9.8 [9.2–10.4]	518	13.4 (12.4–14.5)	13.3 [12.3–14.5]	449	6.8 (6.2–7.4)	6.8 [6.2–7.4]
Not known (N/A)	335	3.2 (2.9–3.6)	2.9 [2.6–3.3]	97	2.5 (2.1–3.1)	2.4 [1.9–2.9]	238	3.6 (3.2–4.1)	3.4 [3.0–3.9]
School type									
Primary school	23	0.2 (0.1–0.3)	0.3 [0.2–0.4]	9	0.2 (0.1–0.5)	0.3 [0.1–0.5]	14	0.2 (0.1–0.4)	0.3 [0.2–0.5]
Secondary school	10,110	96.7 (96.3–97.0)	96.9 [96.5–97.2]	3,754	97.4 (96.8–97.8)	97.5 [96.9–97.9]	6,356	96.3 (95.8–96.7)	96.3 [95.9–96.8]
Further education	327	3.1 (2.8–3.5)	2.9 [2.6–3.2]	93	2.4 (2.0–3.0)	2.3 [1.9–2.9]	234	3.5 (3.1–4.0)	3.4 [3.0–3.8]
School type—gender									
% from mixed	7,345	70.2 (69.3–71.1)	70.7 [69.8–71.6]	2,842	73.7 (72.3)	73.8 [72.3–75.2]	4,503	68.2 (67.1–69.3)	68.1 [66.9–69.2]
School index of multiple deprivation—quintiles									
1^st^ most deprived	492	4.7 (4.3–5.1)	4.8 [4.4–5.3]	119	3.1 (2.6–3.7)	3.4 [2.8–4.1]	373	5.6 (5.1–6.2)	6.1 [5.5–6.7]
2^nd^ quintile	1,886	18.0 (17.3–18.8)	18.2 [17.5–19.0]	561	14.5 (13.5–15.7)	15.3 [14.2–16.6]	1,325	20.1 (19.1–21.0)	20.7 [19.7–21.8]
3^rd^ quintile	1,001	9.6 (9.0–10.1)	9.5 [8.9–10.1]	406	10.5 (9.6–11.5)	10.2 [9.3–11.2]	595	9.0 (8.3–9.7)	8.9 [8.2–9.7]
4^th^ quintile	1,924	18.4 (17.7–19.1)	18.4 [17.6–19.2]	783	20.3 (19.1–21.6)	19.9 [18.7–21.2]	1,141	17.3 (16.4–18.2)	17.1 [16.2–18.0]
5^th^ least deprived	4,822	46.1 (45.1–47.1)	46.1 [45.1–47.1]	1,890	49.0 (47.4–50.6)	48.8 [47.2–50.4]	2,932	44.4 (43.2–45.6)	43.8 [42.6–45.0]
Not known	335	3.2 (2.9–3.6)	2.9 [2.6–3.3]	97	2.5 (2.1–3.1)	2.4 [1.9–2.9]	238	3.6 (3.2–4.1)	3.4 [3.0–3.9]

^a^Weighted to account differences in the distribution of selected sociodemographic variables between the study sample and the target population

^b^’Yes’ includes those who reported having experienced food poverty from ‘once or twice’ to ‘every day’

^c^Excludes 77 (0.7%) observations where data were missing

Clustering at the local authority, school or year group level was not taken into account for the calculation of the confidence intervals

Table 2 shows the unweighted and weighted prevalence of loneliness and self-harm by gender. 18.1% of respondents reported that they often felt lonely (weighted: 16.8%). This was more than twice as common in females. Approximately 8% felt ‘much more’ lonely since lockdown while a further 28% (weighted: 27%) were ‘slightly more’ lonely since lockdown. 18% of respondents scored at the top end (scoring 5 or 6) of the UCLA loneliness items (weighted: 16.6%). 1,452 respondents (13.9%) had self-harmed in their lifetime (weighted: 12.6%). Past year self-harm was reported by 10.8% (weighted: 9.7%) while self-harm during lockdown was reported by 7.5% (weighted: 6.7%), 3.5% (weighted: 3.4%) in males, 14% (weighted: 13.6%) in females.

**Table 2:** Prevalence of loneliness and self-harm by gender, unweighted and weighed proportions with 95% CI

	*Total*			*Males*			*Females*		
	N	Unweighted % (95% CI)	Weighted % [95% CI]^a^	N	% (95% CI)	Weighted % [95% CI]^a^	N	Unweighted % (95% CI)	Weighted % [95% CI]^a^
Loneliness									
Feel lonely									
Never or hardly ever	4,466	42.7 (41.8–43.6)	44.9 [43.9–45.9]	2,134	55.3 (53.8–56.9)	55.6 [54.0–57.2]	2,332	35.3 (34.2–36.5)	35.8 [34.6–37.0]
Some of the time	4,098	39.2 (38.3–40.1)	38.2 [37.3–39.2]	1,318	34.2 (32.7–35.7)	34.0 [32.5–35.6]	2,780	42.1 (40.9–43.3)	41.9 [40.6–43.1]
often	1,896	18.1 (17.4–18.9)	16.8 [16.1–17.6]	404	10.5 (9.6–11.5)	10.4 [9.4–11.6]	1,492	22.6 (21.6–23.6)	22.4 [21.4–23.4]
*Change in loneliness during 1*^*st*^* lockdown*									
Much less lonely	970	9.3 (8.7–9.9)	9.8 [9.2–10.5]	416	10.8 (9.9–11.8)	11.0 [10.0–12.1]	554	8.4 (7.7–9.1)	8.8 [8.1–9.6]
Slightly less	1,292	12.4 (11.7–13.0)	12.5 [11.8–13.1]	474	12.3 (11.3–13.4)	12.5 [11.4–13.6]	818	12.4 (11.6–13.2)	12.5 [11.7–13.3]
Same	3,564	34.1 (33.2–35.0)	34.9 [33.9–35.9]	1,473	38.2 (36.7–39.8)	38.4 [36.8–40.0]	2,091	31.7 (30.6–32.8)	31.9 [30.8–33.1]
Slightly more	2,939	28.1 (27.2–29.0)	27.0 [26.1–27.9]	908	23.6 (22.2–24.9)	23.1 [21.8–24.5]	2,031	30.8 (29.7–31.9)	30.3 [29.2–31.5]
Much more	863	8.2 (7.7–8.8)	7.6 [7.1–8.1]	202	5.2 (4.6–6.0)	5.1 [4.5–5.9]	661	10.0 (9.3–10.8)	9.7 [9.0–10.5]
Missing	832	7.9 (7.5–8.5)	8.2 [7.7–8.8]	383	9.9 (9.0–10.9)	9.9 [9.0–10.9]	449	6.8 (6.2–7.4)	6.8 [6.2–7.4]
*Total score of the two items from UCLA 3-item loneliness scale (N* = *10,269)*									
2 – less lonely	3,281	32.0 (31.1–32.9)	33.6 [32.7–34.6]	1,603	42.4 (40.8–43.9)	42.3 [40.7–44.0]	1,678	25.9 (24.8–27.0)	26.2 [25.1–27.3]
3	2,564	25.0 (24.1–25.8)	25.1 [24.2–26.0]	977	25.8 (24.5–27.2)	25.9 [24.5–27.4]	1,587	24.5 (23.4–25.6)	24.4 [23.3–25.5]
4	2,575	25.1 (24.3–25.9)	24.6 [23.7–25.5]	822	21.7 (20.4–23.1)	21.8 [20.4–23.2]	1,753	27.0 (26.0–28.1)	27.0 [25.9–28.1]
5	1,183	11.5 (10.9–12.2)	10.7 [10.2–11.4]	258	6.8 (6.1–7.7)	6.7 [5.9–7.5]	925	14.3 (13.4–15.1)	14.2 [13.4–15.1]
6 – more lonely	666	6.5 (6.0–7.0)	5.9 [5.5–6.4]	124	3.3 (2.8–3.9)	3.3 [2.7–3.9]	542	8.4 (7.7–8.1)	8.2 [7.6–8.9]
Self-harm									
Lifetime self-harm	1,452	13.9 (13.2–14.6)	12.6 [12.0–13.2]	283	7.3 (6.6–8.2)	7.1 [6.3–8.0]	1,169	17.7 (16.8–15.6)	17.2 [16.3–18.2]
Past year self-harm	1,129	10.8 (10.2–11.4)	9.7 [9.2–10.3]	204	5.3 (4.6–6.0)	5.2 [4.5–5.9]	925	14.0 (13.2–14.9)	13.6 [12.8–14.4]
Past six months	879	8.4 (7.9–9.0)	7.5 [7.0–8.0]	152	3.9 (3.4–4.6)	3.8 [3.3–4.5]	727	11.0 (10.3–11.8)	10.6 [9.9–11.4]
Self-harm during 1^st^ UK lockdown	787	7.5 (7.0–8.1)	6.7 [6.3–7.2]	135	3.5 (3.0–4.1)	3.4 [2.8–4.0]	652	9.9 (9.2–10.6)	9.6 [8.9–10.3]

^a^Weighted to account for differences in the distribution of selected sociodemographic variables between the study sample and the target population

Clustering at the local authority, school or year group level was not taken into account for the calculation of the confidence intervals

We estimated the associations between indicators of loneliness and self-harm (Table 3). Models were weighted as above. Controlling for demographic characteristics, indicators of socioeconomic deprivation and mental health, adolescents who reported feeling lonely ‘often’ or ‘sometimes’ were more likely to report self-harm during lockdown relative to their peers who reported ‘never’ or ‘hardly ever’ feeling lonely [often lonely: adjusted odds ratio (aOR) 2.8, 95% CI 2.1–3.9, *p* < 0.0001; sometimes lonely: aOR 2.2, 95% CI 1.5–3.2, *p* < 0.0001]. A higher score on the loneliness measure was associated with greater odds of reporting self-harm during lockdown, after adjusting for measured confounders. Furthermore, exacerbation in loneliness during lockdown was associated with an increase in the odds of self-harm during this time. Respondents who reported feeling more lonely during the period of lockdown were twice as likely to also report self-harm during this time, compared to adolescents who felt much less lonely during this time (Table 3).

**Table 3:** The associations of indicators of loneliness and self-harm, Odds ratio and 95% CI

	*Odds ratio (95% CI)*				
	N [n self-harmed]	Unadjusted	p value	Adjusted^a^	P value
Self-harm during 1^st^lockdown^b^ (*N* = 10,432 with information on lockdown self-harm)					
*Feel lonely*					
* Never or hardly ever*	4,463 [47]	1.0		1.0	
* Sometimes*	4,087 [292]	6.9 (4.1–11.6)	< 0.0001	2.2 (1.5–3.2)	< 0.0001
* Often*	1,882 [448]	28.3 (19.9–40.3)	< 0.0001	2.8 (2.1–3.9)	< 0.0001
*UCLA loneliness score*^c^	10,241 [780]	2.2 (2.1–2.3)	< 0.0001	1.2 (1.1–1.3)	< 0.0001
*Change in loneliness during lockdown*^b^ *(N* = *9,603 with information on self-harm and change in loneliness during lockdown*)					
*Much less lonely*	968 [30]	1.0		1.0	
*Slightly less*	1,289 [71]	2.1 (1.1–4.0)	0.04	2.0 (1.0–4.0)	0.07
*Same*	3,557 [144]	1.6 (0.8–3.3)	0.24	1.6 (0.7–1.4)	0.25
*Slightly more*	2,932 [313]	4.4 (4.4–8.0)	< 0.0001	2.1 (1.1–3.9)	0.03
*Much more*	857 [212]	12.2 (5.8–25.7)	< 0.0001	2.4 (1.1–5.0)	0.02
*Continuous*	9,603 [770]	1.033 (1.028–1.037)	< 0.0001	1.007 (1.004–1.011)	< 0.0001

^a^Adjusted for gender, age (year group), food poverty, free school meals, child birth place, symptoms of anxiety and depression, history of receiving mental health support

^b^In the UK 1^st^ lockdown: from 23^rd^ March to June 2020

^c^Score is based on two items from the UCLA loneliness scale

Clusters: local authority; school; year group

The analysis is weighted to account for differences in the distribution of selected sociodemographic variables between the study sample and the target population

## Discussion

We aimed to estimate the prevalence of loneliness and self-harm in a sample of 10,460 12–18 year-olds from the south of England during the early stages of the COVID-19 pandemic and to investigate the associations between loneliness (its intensity and change during lockdown) and self-harm using data from a cross-sectional school survey.

17% of adolescents surveyed reported that they often felt lonely, a higher proportion than figures reported in other surveys from 2017 (9.5%) (Yang et al., 2020) and 2018 (11.3%) (Office for National Statistics, 2018a); although these studies are not directly comparable because they were conducted on different samples. To allow for comparison, we recalculated the prevalence of loneliness in respondents aged 12–15 years, an age range more similar to that reported by ONS (Office for National Statistics, 2018a). The prevalence of loneliness in 12–15 year-olds in our survey was 15%. Furthermore, findings from a retrospective survey in Scotland have shown an increase in the prevalence of loneliness among 12–17 year-olds from 9% before lockdown to 28% during lockdown (Generation Scotland, 2020). Similarly, data from the YouGov survey showed that 41% of the children and young persons surveyed reported that they were lonelier than before lockdown (Barnardo’s, 2020), a figure which is broadly in line with the OxWell findings (35% of respondents). Taken together, the data suggest that a substantial proportion of adolescents frequently felt lonely during the time of national lockdown and that loneliness had intensified during this period for many adolescents. These findings concur with concerns expressed in the scientific literature about the potential negative effect of the pandemic and the resulting measures to curb the spread of the virus on the mental health and well-being of young persons (Loades et al., 2020) although not all studies are consistent with the above (Houghton et al., 2022; Myhr et al., 2021).

Seven percent of respondents in our survey reported that they had self-harmed during lockdown, with almost twice that reporting that they had self-harmed at some point in their life. These figures highlight a sizable group of adolescents who have been experiencing significant distress and were at risk of further self-harm and probably needed support which might have been lost or significantly disrupted during this challenging time. Importantly, the true prevalence of self-harm is likely to have been even higher as case-ascertainment in this study was contingent on a response to an open-ended question about the method of self-harm and responding was optional (see Methods).

Our finding that adolescents who reported more intense loneliness were more likely to have self-harmed, with those experiencing increase in loneliness during lockdown showing increased risk of self-harm during the same period adds to the body of literature which shows a positive association between indicators of loneliness and suicidal thoughts and behaviour in young persons; although it is worth noting that not all studies have demonstrated an association between loneliness and self-harm (Calati et al., 2019; McClelland et al., 2020). The findings from this study are also in keeping with theories of suicidal behaviour (Joiner, 2005; Klonsky & May, 2015; Van Orden et al., 2010) which maintain that social ties and sense of belongingness play a key role in suicidal behaviour. It is plausible that the unprecedent disruption to adolescents’ usual support networks contributed to increased loneliness and significant distress which, in the presence of additional risk factors, increased their self-harm, especially given that in young persons, friends are a key source of support for mental health problems (Migliorini et al., 2021) and self-harm (Geulayov et al., 2022).

Our finding that change in loneliness was related to the risk of self-harm is novel. It points to a need to better understand how changes in loneliness in response to changes in individuals’ circumstances may be followed by changes to individuals’ distress and self-harm. Furthermore, loneliness is a potentially modifiable factor so studying how to support those experiencing loneliness, and whether structural and environmental changes can help reduce loneliness, could be an important component of future interventions and warrants research attention and consideration by all providers to this age group.

### Strengths and limitations

The findings are from a large school-based survey conducted across 11 local authorities in south England. The survey does not collect explicit identifiers, which is likely to facilitate disclosure of behaviours and experiences that may be associated with stigma. Indeed, preliminary findings from this survey show that individuals who have self-harmed reported that they would respond differently had identifying information been sought (Mansfield et al., 2021, personal communication).

The study has a number of limitations. Measures of loneliness and self-harm were taken at a single point in time and so we cannot be certain about the direction of association. Further work using prospective designs is needed to better understand the nature of the relationship between loneliness and self-harm and whether or not changes in loneliness over time are related to changes in suicidal thoughts and self-harm. The survey was completed primarily while pupils were at home (due to the national lockdown). Differences in the propensity of individuals to participate in the survey, and access to an electronic devise required to complete the survey, may have introduced some bias. However, some pupils considered vulnerable or ‘at-risk' were able to attend school during this period and had accessed the survey whilst at school. We address the above limitations by deriving weights which include sociodemographic variables and indicators of deprivation using the UK Census data for the target catchment areas, although applying post-stratification weights may not fully account for the differences between the sample and the target population. This limitation is common to studies conducted in the early phases of the pandemic (Pierce et al., 2020) and the results should, therefore, be interpreted with caution. Furthermore, we surveyed a population from schools across the south of England so the findings may not be generalisable to other parts of the country. A nationally representative random sample would be necessary to be able to obtain more accurate estimates of the prevalence of self-harm which can be generalised to the country as a whole.

It is important to consider school-based approaches when addressing loneliness and self-harm in adolescent populations. Schools can play a vital role in preventing and alleviating loneliness as well as in addressing self-harm. Schools may address adolescents’ loneliness by increasing opportunities for social contact through structured and unstructured activities. Such activities can provide opportunities for pupils to meet one another through shared tasks, interests or pursuits which may also improve their social skills and enhance a sense of belonging to their school community. Schools can also be an avenue for delivering self-harm interventions and there is some evidence that school-based interventions may be beneficial. The Saving and Empowering Young Lives in Europe (SEYLE) (Wasserman et al., 2015) and the Good Behaviour Game study (Katz et al., 2013; Wilcox et al., 2008) are interventions that have shown some benefit in schools although their positive findings have yet to be replicated in the UK context.

Addressing loneliness and promoting belonging in schools may also help address the impact of the pandemic on loneliness itself as well as on emotional difficulties and self-harm. These opportunities, often best achieved whilst at school, might need to be prioritised, even over academic activities, to try and address social networks as well as the mental health of adolescents. Determining what type of interventions adolescents find most acceptable to support them in tackling loneliness will likely be an essential component of many interventions and strategies moving forward. Placing greater emphasis on the role of ‘quality social connections’ (Dewa et al., 2020), a concept that has been investigated as an ‘active ingredient’ in interventions for mental health problems in young people holds promise and its role in addressing self-harm in the school context needs to be evaluated.

## Conclusions

This study showed that one in six adolescents experienced intense feelings of loneliness in the early stages of the pandemic, with one in three feeling lonelier than before. One in 15 adolescents had self-harmed during the first lockdown. Self-harm was twice as common in adolescents who report experiencing intense loneliness, and feeling lonelier than before lockdown increased the risk of self-harm during lockdown. The findings highlight the importance of addressing both loneliness and self-harm in adolescent school pupils as part of an effort to improve their well-being and reduce their risk of self-harm. It is also necessary to continue to monitor loneliness and self-harm in young persons in the community in the longer term as the pandemic and its resultant social and financial impacts unfold. Understanding how loneliness and self-harm may co-vary can be important for future self-harm reduction strategies in young persons.

## Supplementary Information

Below is the link to the electronic supplementary material.

Supplementary file1 (DOCX 39 KB)

## Data Availability

Fully anonymous extracts of the data can be provided to academic research collaborators upon reasonable request, following a careful review process by the research team. The data are not publicly available due to ethical and information governance restrictions.

## Code availability

The code used in this analysis is available on request from the authors.

## References

[CR1] Barnardo’s. (2020). *Generation lockdown: a third of children and young people experience increased mental health difficulties*. Retrieved 14th June from https://www.barnardos.org.uk/news/generation-lockdown-third-children-and-young-people-experience-increased-mental-health

[CR2] Borschmann R, Becker D, Coffey C, Spry E, Moreno-Betancur M, Moran P, Patton GC (2017). 20-year outcomes in adolescents who self-harm: A population-based cohort study. Lancet Child Adolesc Health.

[CR3] Calati R, Ferrari C, Brittner M, Oasi O, Olie E, Carvalho AF, Courtet P (2019). Suicidal thoughts and behaviors and social isolation: A narrative review of the literature. Journal of Affective Disorders.

[CR4] Carr MJ, Steeg S, Webb RT, Kapur N, Chew-Graham CA, Abel KM, Hope H, Pierce M, Ashcroft DM (2021). Effects of the COVID-19 pandemic on primary care-recorded mental illness and self-harm episodes in the UK: A population-based cohort study. Lancet Public Health.

[CR5] Child Outcomes Research Consortium (CORC). (2020). *Revised Children’s Anxiety and Depression Scales*. Retrieved September 1st 2021 from https://www.corc.uk.net/outcome-experience-measures/revised-childrens-anxiety-and-depression-scale-rcads/

[CR6] Chorpita BF, Yim L, Moffitt C, Umemoto LA, Francis SE (2000). Assessment of symptoms of DSM-IV anxiety and depression in children: A revised child anxiety and depression scale. Behaviour Research and Therapy.

[CR7] Department for Digital Culture Media and Sport. (2020). *Wellbeing and Loneliness - Community Life Survey 2019/20*. Retrieved 14th June from https://www.gov.uk/government/statistics/community-life-survey-201920-wellbeing-and-loneliness/wellbeing-and-loneliness-community-life-survey-201920

[CR8] Dewa, L. H., Lawrance, E., Roberts, L., Brooks-Hall, E., Ashrafian, H., Fontana, G., & Aylin, P. (2021). Quality social connection as an active ingredient in digital interventions for young people with depression and anxiety: Systematic scoping review and meta-analysis. *Journal of Medical Internet Research*, *23*(12), e26584. 10.2196/26584 10.2196/26584 PMC8726025 34927592

[CR9] Ebesutani C, Reise SP, Chorpita BF, Ale C, Regan J, Young J, Higa-McMillan C, Weisz JR (2012). The Revised Child Anxiety and Depression Scale-Short Version: Scale reduction via exploratory bifactor modeling of the broad anxiety factor. Psychological Assessment.

[CR10] Ellis WE, Dumas TM, Forbes LM (2020). Physically Isolated but Socially Connected: Psychological Adjustment and Stress Among Adolescents During the Initial COVID-19 Crisis. Canadian Journal of Behavioural Science-Revue Canadienne Des Sciences Du Comportement.

[CR11] Evans E, Hawton K, Rodham K (2004). Factors associated with suicidal phenomena in adolescents: A systematic review of population-based studies. Clinical Psychology Review.

[CR12] Ferrey AE, Hughes ND, Simkin S, Locock L, Stewart A, Kapur N, Gunnell D, Hawton K (2016). The impact of self-harm by young people on parents and families: A qualitative study. British Medical Journal Open.

[CR13] Generation Scotland. (2020). *TeenCovidLife*. Retrieved 14th June from https://www.ed.ac.uk/generation-scotland/what-have-we-found/latest-news/teencovidlife-one

[CR14] Geulayov, G., Borschmann, R., Mansfield, K. L., Hawton, K., Moran, P., & Fazel, M. (2022). Utilization and acceptability of formal and informal support for adolescents following self-harm before and during the first COVID-19 lockdown: Results from a large-scale english schools survey. *Frontiers in Psychiatry*, *13*, 881248. 10.3389/fpsyt.2022.881248 10.3389/fpsyt.2022.881248 PMC9263724 35815012

[CR15] Geulayov G, Casey D, Bale L, Brand F, Clements C, Farooq B, Kapur N, Ness J, Waters K, Tsiachristas A, Hawton K (2019). Suicide following presentation to hospital for non-fatal self-harm in the Multicentre Study of Self-harm: A long-term follow-up study. Lancet Psychiatry.

[CR16] Geulayov G, Casey D, McDonald KC, Foster P, Pritchard K, Wells C, Clements C, Kapur N, Ness J, Waters K, Hawton K (2018). Incidence of suicide, hospital-presenting non-fatal self-harm, and community-occurring non-fatal self-harm in adolescents in England (the iceberg model of self-harm): A retrospective study. Lancet Psychiatry.

[CR17] Gillies D, Christou MA, Dixon AC, Featherston OJ, Rapti I, Garcia-Anguita A, Villasis-Keever M, Reebye P, Christou E, Al Kabir N, Christou PA (2018). Prevalence and Characteristics of Self-Harm in Adolescents: Meta-Analyses of Community-Based Studies 1990–2015. Journal of the American Academy of Child and Adolescent Psychiatry.

[CR18] Hawton K, Bale L, Brand F, Townsend E, Ness J, Waters K, Clements C, Kapur N, Geulayov G (2020). Mortality in children and adolescents following presentation to hospital after non-fatal self-harm in the Multicentre Study of Self-harm: A prospective observational cohort study. Lancet Child Adolesc Health.

[CR19] Hawton K, Bergen H, Kapur N, Cooper J, Steeg S, Ness J, Waters K (2012). Repetition of self-harm and suicide following self-harm in children and adolescents: Findings from the Multicentre Study of Self-harm in England. Journal of Child Psychology and Psychiatry.

[CR20] Hawton K, Harriss L, Hall S, Simkin S, Bale E, Bond A (2003). Deliberate self-harm in Oxford, 1990–2000: A time of change in patient characteristics. Psychological Medicine.

[CR21] Hawton K, Hill NTM, Gould M, John A, Lascelles K, Robinson J (2020). Clustering of suicides in children and adolescents. Lancet Child Adolesc Health.

[CR22] Hawton K, Rodham K, Evans E, Weatherall R (2002). Deliberate self-harm in adolescents: Self report survey in schools in England. BMJ.

[CR23] Houghton S, Kyron M, Hunter SC, Lawrence D, Hattie J, Carroll A, Zadow C (2022). Adolescents' longitudinal trajectories of mental health and loneliness: The impact of COVID-19 school closures. Journal of Adolescence.

[CR24] Joiner T (2005). Why people die by suicide.

[CR25] Jollant, F., Roussot, A., Corruble, E., Chauvet-Gelinier, J. C., Falissard, B., Mikaeloff, Y., & Quantin, C. (2021). Hospitalization for self-harm during the early months of the COVID-19 pandemic in France: A nationwide retrospective observational cohort study. *Lancet Reg Health Eur*, *6*, 100102. 10.1016/j.lanepe.2021.100102 10.1016/j.lanepe.2021.100102 PMC8454825 34557830

[CR26] Katz C, Bolton SL, Katz LY, Isaak C, Tilston-Jones T, Sareen J, Swampy Cree Suicide Prevention, T. (2013). A systematic review of school-based suicide prevention programs. Depress Anxiety.

[CR27] Klonsky ED, May AM (2015). The Three-Step Theory (3ST): A New Theory of Suicide Rooted in the "Ideation-to-Action" Framework. International Journal of Cognitive Therapy.

[CR28] Loades ME, Chatburn E, Higson-Sweeney N, Reynolds S, Shafran R, Brigden A, Linney C, McManus MN, Borwick C, Crawley E (2020). Rapid Systematic Review: The Impact of Social Isolation and Loneliness on the Mental Health of Children and Adolescents in the Context of COVID-19. J Am Acad Child Adolesc Psychiatry.

[CR29] Madge N, Hewitt A, Hawton K, de Wilde EJ, Corcoran P, Fekete S, Van Heeringen K, De Leo D, Ystgaard M (2008). Deliberate self-harm within an international community sample of young people: comparative findings from the Child and Adolescent Self-harm in Europe (CASE) Study. Journal of Child Psychology and Psychiatry and Allied Disciplines.

[CR30] Mansfield, K. L., Newby, D., Soneson, E., Vaci, N., , Jindra, C., Geulayov, G., Gallacher, J., & Fazel, M. (2021). COVID‐19 partial school closures and mental health problems: A cross‐sectional survey of 11,000 adolescents to determine those most at risk. *JCPP Advances,* e12021. 10.1111/jcv2.12021 10.1002/jcv2.12021 PMC8420157 34514466

[CR31] Mars B, Heron J, Crane C, Hawton K, Lewis G, Macleod J, Tilling K, Gunnell D (2014). Clinical and social outcomes of adolescent self harm: Population based birth cohort study. BMJ.

[CR32] McClelland H, Evans JJ, Nowland R, Ferguson E, O'Connor RC (2020). Loneliness as a predictor of suicidal ideation and behaviour: A systematic review and meta-analysis of prospective studies. Journal of Affective Disorders.

[CR33] McMahon EM, Keeley H, Cannon M, Arensman E, Perry IJ, Clarke M, Chambers D, Corcoran P (2014). The iceberg of suicide and self-harm in Irish adolescents: A population-based study. Social Psychiatry and Psychiatric Epidemiology.

[CR34] Migliorini C, Lam DS, Harvey C (2021). Supporting family and friends of young people with mental health issues using online technology: A rapid scoping literature review. Early Intervention in Psychiatry.

[CR35] Moran P, Coffey C, Romaniuk H, Olsson C, Borschmann R, Carlin JB, Patton GC (2012). The natural history of self-harm from adolescence to young adulthood: A population-based cohort study. The Lancet.

[CR36] Morgan C, Webb RT, Carr MJ, Kontopantelis E, Green J, Chew-Graham CA, Kapur N, Ashcroft DM (2017). Incidence, clinical management, and mortality risk following self harm among children and adolescents: Cohort study in primary care. BMJ.

[CR37] Myhr A, Naper LR, Samarawickrema I, Vesterbekkmo RK (2021). Impact of COVID-19 Pandemic Lockdown on Mental Well-Being of Norwegian Adolescents During the First Wave-Socioeconomic Position and Gender Differences. Frontiers in Public Health.

[CR38] O’Connor, R. C., Wetherall, K., Cleare, S., McClelland, H., Melson, A. J., Niedzwiedz, C. L., O’Carroll, R. E., O’Connor, D. B., Platt, S., Scowcroft, E., Watson, B., Zortea, T., Ferguson, E., & Robb, K. A. (2020). Mental health and well-being during the COVID-19 pandemic: Longitudinal analyses of adults in the UK COVID-19 Mental Health & Wellbeing study. *British Journal of Psychiatry,**1–8*,. 10.1192/bjp.2020.212 10.1192/bjp.2020.212 PMC7684009 33081860

[CR39] Office for National Statistics. (2018a). *Children’s and young people’s experiences of loneliness: 2018a*. Retrieved 14th June from https://www.file:///C:/Users/galitg/Downloads/Children%20s%20and%20young%20people%20s%20experiences%20of%20loneliness%202018a.pdf

[CR40] Office for National Statistics. (2018b). *Testing of loneliness questions in surveys*. Retrieved 14th June from https://www.file:///C:/Users/galitg/Downloads/Testing%20of%20loneliness%20questions%20in%20surveys%20(1).pdf

[CR41] Olfson M, Wall M, Wang S, Crystal S, Bridge JA, Liu SM, Blanco C (2018). Suicide After Deliberate Self-Harm in Adolescents and Young Adults. Pediatrics.

[CR42] Patalay P, Gage SH (2019). Changes in millennial adolescent mental health and health-related behaviours over 10 years: A population cohort comparison study. International Journal of Epidemiology.

[CR43] Perlman, D., Peplau, L.A. (1981). Toward a Social Psychology of Loneliness. In S. W. Duck, Gilmour, R. (Ed.) *Personal Relationships in Disorder*. Academic Press.

[CR44] Pierce M, McManus S, Jessop C, John A, Hotopf M, Ford T, Hatch S, Wessely S, Abel KM (2020). Says who? The significance of sampling in mental health surveys during COVID-19. Lancet Psychiatry.

[CR45] Russell D, Peplau LA, Cutrona CE (1980). The revised UCLA Loneliness Scale: Concurrent and discriminant validity evidence. Journal of Personality and Social Psychology.

[CR46] Russell DW (1996). UCLA Loneliness Scale (Version 3): Reliability, validity, and factor structure. Journal of Personality Assessment.

[CR47] Van Orden KA, Witte TK, Cukrowicz KC, Braithwaite SR, Selby EA, Joiner TE (2010). The interpersonal theory of suicide. Psychological Review.

[CR48] Wasserman, D., Hoven, C. W., Wasserman, C., Wall, M., Eisenberg, R., Hadlaczky, G., Kelleher, I., Sarchiapone, M., Apter, A., Balazs, J., Bobes, J., Brunner, R., Corcoran, P., Cosman, D., Guillemin, F., Haring, C., Iosue, M., Kaess, M., Kahn, J. P., … & Carli, V. (2015). School-based suicide prevention programmes: The SEYLE cluster-randomised, controlled trial. *Lancet,**385*(9977), 1536–1544. 10.1016/S0140-6736(14)61213-7 10.1016/S0140-6736(14)61213-7 25579833

[CR49] Wilcox HC, Kellam SG, Brown CH, Poduska JM, Ialongo NS, Wang W, Anthony JC (2008). The impact of two universal randomized first- and second-grade classroom interventions on young adult suicide ideation and attempts. Drug and Alcohol Dependence.

[CR50] Yang, K. M., Petersen, K. J., & Qualter, P. (2020). Undesirable social relations as risk factors for loneliness among 14-year-olds in the UK: Findings from the Millennium Cohort Study. *International Journal of Behavioral Development*. Artn 0165025420965737. 10.1177/0165025420965737

