# Supplementary material for: Loneliness and self-harm in adolescents during the first national COVID-19 lockdown: results from a survey of 10,000 secondary school pupils in England
Source: Curr Psychol. 2022 Sep 15:1–12. Online ahead of print. doi: 10.1007/s12144-022-03651-5 (PMC9476392; doi:10.1007/s12144-022-03651-5)
Supplement: Supplementary file 1 — Supplementary file1 (DOCX 39 KB) [file 12144_2022_3651_MOESM1_ESM.docx]

**­Supplementary material**

**Fig. S1** Sample selection process

Home/school web-based survey: June-July 2020

Year 8-13 pupils (n=14,352) from 91 schools (84 secondary schools and 7 further education colleges) across 11 local authorities in Oxfordshire, Buckinghamshire, Gloucestershire, South Gloucestershire, Wiltshire, Bristol, North Somerset, Slough, Reading, Windsor and Maidenhead, Bracknell Forest enrolled to take part

Excluded due to insufficient information:

884: incomplete or inconsistent responses

1703: spent less than 10 minutes completing the survey

11,765 pupils included in sample

1,305 observations excluded from the analysis due to missing data on one or more key variables or because the pupil was older than 18 years:

84: Gender unknown

68: age not known (n=31), age>18 years (n=37)

1077: Self-harm status unknown

461: No information about loneliness

*Categories are not mutually exclusive

10,460 pupils in year 8-13 from 90 schools across 11 local authorities included in analysis

Table S1: Self-harm questionnaire

|  | Item | Response options | *Item as appearing in the text* |
| --- | --- | --- | --- |
| 1 | Have you ever deliberately self-harmed (for example by taking an overdose or deliberately injuring yourself in some other way)? | Yes/ No/ Not sure what this means | *Ever self-harmed* |
| 2 | Have you ever deliberately injured yourself in some way? | Never-Once or twice-A few times-Weekly -Daily | *Ever self-injured* |
| 3 | Has this happened during lockdown? | Never-Once or twice-Monthly-Weekly-Most days | *Self-injured during lockdown* |
| 4 | When did you last self-harm? | In the last week-In the last month-in the past 3-6 months-6 months to a year ago-Over a year ago | *Last self-injury* |
| 5 | Have you ever deliberately taken an overdose (e.g. of pills or other medication)? | Never Yes - Once Yes - On more than one occasion | *Ever self-poisoned* |
| 6 | Has this happened during lockdown? | Never Yes - Once Yes - On more than one occasion | *Self-poisoned during lockdown* |
| 7 | When did you last take an overdose? | In the last week-In the last month-in the past 3-6 months-6 months to a year ago-Over a year ago | *Last self-poisoned* |
| 8 | The last time this happened, in what way did you self-harm/ overdose? | [Free text response with 100 character limit] | *Description of self-harm act* |
